# Fuzzy Logic and Its Application in Football Team Ranking

**DOI:** 10.1155/2014/291650

**Published:** 2014-06-16

**Authors:** Wenyi Zeng, Junhong Li

**Affiliations:** College of Information Science and Technology, Beijing Normal University, Beijing 100875, China

## Abstract

Fuzzy set theory and fuzzy logic are a highly suitable and applicable basis for developing knowledge-based systems in physical education for tasks such as the selection for athletes, the evaluation for different training approaches, the team ranking, and the real-time monitoring of sports data. In this paper, we use fuzzy set theory and apply fuzzy clustering analysis in football team ranking. Based on some certain rules, we propose four parameters to calculate fuzzy similar matrix, obtain fuzzy equivalence matrix and the ranking result for our numerical example, *T*_7_, *T*_3_, *T*_1_, *T*_9_, *T*_10_, *T*_8_, *T*_11_, *T*_12_, *T*_2_, *T*_6_, *T*_5_, *T*_4_, and investigate four parameters sensitivity analysis. The study shows that our fuzzy logic method is reliable and stable when the parameters change in certain range.

## 1. Introduction

In recent years, computational intelligence has been used to solve many complex problems by developing intelligent systems. And fuzzy logic has proved to be a powerful tool for decision-making systems, such as expert systems and pattern classification systems. Fuzzy set theory has already been applied in some physical expert systems.

In traditional rule-based approach, knowledge is encoded in the form of antecedent-consequent structure. When new data is encountered, it is matched to the antecedents clauses of each rule, and those rules, where antecedents match a datum exactly, are fired; then we establish the consequent clauses. This process continues until the desired conclusion is reached. In the past decade, fuzzy logic has proved to be wonderful tool for intelligent systems in physical training. Some examples of using fuzzy logic to develop fuzzy intelligent systems are fuzzy systems in their microprocessors, fuzzy control of the subway system, fuzzy washing machines, fuzzy cameras, and camcorders that map image data to lens settings.

Fuzzy sets introduced by Zadeh [1] have been found to be an important tool to deal with vagueness and uncertainty. Over the last decades, many researchers have investigated the fuzzy set theory and applied it to various fields including decision making, logic programming, medical diagnosis, pattern recognition, fuzzy inference, and fuzzy control. Clustering analysis is a fundamental and important method in statistical data analysis which has been widely investigated and applied in a variety of fields such as pattern recognition, information retrieval, microbiology analysis, and data mining [2–5]. Wang [6] introduced the concepts, including the degree of fuzzy similarity, fuzzy similarity matrix, and fuzzy equivalence matrix, and then gave a procedure for transforming the fuzzy similarity matrix into the fuzzy equivalence matrix. After that, a clustering technique of fuzzy sets was extensively applied in many fields [2, 6–8] on the basis of the *λ*-cutting matrix of the fuzzy equivalence matrix. Since then, some researchers have investigated this topic and have obtained some meaningful conclusions. For example, Ansari et al. [9] applied the fuzzy clustering analysis method in identification of hidden patterns among historical and instrumental seismic catalog of Iran. Benati [10] investigated categorical data fuzzy clustering and applied it in the analysis of local search heuristics. Yang and Watada [11] applied fuzzy clustering analysis method to return appropriate information for user queries, data annotation, and underlying ontology. Li [12] and He et al. [13] investigated fuzzy clustering method based on perturbation, respectively. Xie and Beni [14] investigated the validity measure for fuzzy clustering. Zhang et al. [15] investigated the cluster validity index for fuzzy clustering analysis. Furthermore, Dunn [16] proposed the fuzzy c-means (FCM) clustering algorithm and Bezdek [2] extended the FCM method and made the FCM method to become the most well-known in fuzzy clustering analysis. Recently, Pan [17] combined fuzzy common mapping with fuzzy clustering approach to perform clustering effect analysis. Bozkir and Sezer [18] developed a desktop software, FUAT, and used it to analyze and investigate different aspects in order to obtain and inform possible natural cluster number.

Ranking in the physical matches is an important and interesting topic. But the ranking becomes complicated because of a large amount of teams, data missing of the sport teams, and some other reasons. Aimed at the ranking problem, how to construct a mathematical model is an interesting topic. In this paper, we merge ranking problem with fuzzy clustering analysis. According to the queuing principle, if we choose one objective to be the best (or worst), then the next should be the second best (or worst) which belongs to this type that the first objective is in, and the others are similar.

The organization of our work is as follows. In Section 2, we recall some preliminaries for fuzzy clustering analysis. In Section 3, we give an application of fuzzy clustering analysis in ranking. The final section is conclusion.

## 2. Some Notions

Throughout this paper, we use *X* = {*x*_1_, *x*_2_,…, *x*_*n*_} to denote a set of *n* objects. *r*_*ij*_ represents the degree of fuzzy similarity between the objects *x*_*i*_ and *x*_*j*_.

For clarity, some definitions and notations are listed as follows.

**Definition 1 .**

A binary operation ∧:[0, 1]×[0, 1]→[0, 1] is called a *t*-norm if it satisfies the following properties:

1. (identity) 1∧*x* = *x*;
2. (commutativity) *x*∧*y* = *y*∧*x*;
3. (associativity) *x*∧(*y*∧*z*) = (*x*∧*y*)∧*z*;
4. (monotonicity) If *w* ≤ *x* and *y* ≤ *z* then *w*∧*y* ≤ *x*∧*z*.

**Definition 2 .**

A binary operation ∨:[0, 1]×[0, 1]→[0, 1] is called a *t*-conorm if it satisfies the following properties:

1. (identity) 0∨*x* = *x*;
2. (commutativity) *x*∨*y* = *y*∨*x*;
3. (associativity) *x*∨(*y*∨*z*) = (*x*∨*y*)∨*z*;
4. (monotonicity) If *w* ≤ *x* and *y* ≤ *z* then *w*∨*y* ≤ *x*∨*z*.

Here are three basic examples of *t*-norms and *t*-conorms which are often used for reasoning in fuzzy logic systems:

1. *a*∧*b* = min⁡ (*a*, *b*), the corresponding *t*-conorm can be obtained by using de Morgan laws: *a*∨*b* = max⁡ (*a*, *b*);
2. *a*∧*b* = *a* · *b*, the corresponding *t*-conorm is *a*∨*b* = *a* + *b* − *a* · *b*;
3. *a*∧*b* = max⁡ (*a* + *b* − 1, 0), the corresponding *t*-conorm is *a*∨*b* = min⁡ (*a* + *b*, 1).

**Definition 3 .**

If *A* = (*a*_*ij*_)_*m*×*n*_ is a fuzzy matrix such that 0 ≤ *a*_*ij*_ ≤ 1 for *i* = 1,2,…, *m*, *j* = 1,2,…, *n*, then we call *A*_*λ*_ = (*a*_*ij*_^(*λ*)^)_*m*×*n*_ the *λ*-cutting matrix of the fuzzy matrix *A*, where

(1) $$\begin{array}{c} a_{ij}^{\left(\lambda \right)}=\{\begin{array}{cc} 0, & \text{if}\;a_{ij}<\lambda ,\\ 1, & \text{if}\;a_{ij}\geq \lambda \end{array} \end{array}$$

and *λ* is the confidence level with *λ* ∈ [0, 1].

**Definition 4 .**

Let *A* = (*a*_*ij*_)_*m*×*n*_ and *B* = (*b*_*ij*_)_*n*×*p*_ be *m* × *n* and *n* × *p* fuzzy matrixes, respectively; then *A*∘*B* is *m* × *p* fuzzy matrix and is called composition matrix of the *m* × *n* fuzzy matrix *A* and the *n* × *p* fuzzy matrix *B*, where “∘” is called the composition operation of fuzzy matrixes, written by *C* = *A*∘*B* = (*c*_*ij*_)_*m*×*p*_ and *c*_*ij*_ is computed as follows:

(2) $$\begin{array}{c} c_{ij}=\overset{n}{\underset{k=1}{⋁}}\left(a_{ik}\wedge b_{kj}\right),\;i=1,2,\ldots ,m,\;\;j=1,2,\ldots ,p. \end{array}$$

If *A* = *B* = (*a*_*ij*_)_*n*×*n*_, specially, then we denote *A* ^2^ = *A*∘*A*. Generally, we have *A* ^*n*^ = *A* ^*n*−1^∘*A* if *n* is a nonnegative integer.

**Definition 5 .**

A fuzzy matrix *A* = (*a*_*ij*_)_*n*×*n*_ is reflexive if *a*_*ii*_ = 1 for 1 ≤ *i* ≤ *n*. A fuzzy matrix *A* = (*a*_*ij*_)_*n*×*n*_ is symmetric if *a*_*ij*_ = *a*_*ji*_ for 1 ≤ *i*, *j* ≤ *n*. A fuzzy matrix *A* = (*a*_*ij*_)_*n*×*n*_ is max-min transitive if *a*_*ij*_ ≥ ⋁_*k*=1_^*n*^  (*a*_*ik*_∧*a*_*kj*_), where “∨” and “∧” stand for max and min operation, respectively.

**Definition 6 .**

A fuzzy matrix is fuzzy similarity matrix when it is reflexive and symmetric. A fuzzy matrix is fuzzy equivalence matrix when it is reflexive, symmetric, and max-min transitive.

**Definition 7 .**

Fuzzy matrix *B* is called the max-min transitive closure of fuzzy matrix *A* if fuzzy matrix *B* includes fuzzy matrix *A* and fuzzy matrix *B* satisfies the following properties: (1) fuzzy matrix *B* is max-min transitive; (2) fuzzy matrix *B* is included by any fuzzy matrix which includes fuzzy matrix *A* and satisfies max-min transitivity.

Known by the above definitions, we can get the following conclusions.

**Theorem 1 (see [6]).**

For a given fuzzy matrix *R* = (*r*_*ij*_)_*n*×*n*_, its max-min transitive closure R* is computed as follows:

(3) $$\begin{array}{c} R^{*}=R\cup R^{2}\cup \cdots \cup R^{n}. \end{array}$$

**Theorem 2 (see [6]).**

For a given fuzzy similarity matrix *R* = (*r*_*ij*_)_*n*×*n*_, there exists the smallest nonnegative integer *k*  (*k* ≤ *n*), such that R* = *R* ^*k*^ and for every nonnegative integer *l*  (*l* > *k*), we have *R* ^*l*^ = *R* ^*k*^, and R* = *R* ^*k*^ is a fuzzy equivalence matrix.

Therefore, known by the above-mentioned Theorem 2, then after the finite times of compositions, we have

(4) $$\begin{array}{c} R⟶R^{2}⟶R^{4}⟶\cdots ⟶R^{2^{k}}⟶\cdots \end{array}$$

and there must exist a positive integer *k* such that R* = *R* ^2^*k*^^ = *R* ^2^*k*+1^^ is a fuzzy equivalence matrix.

Known by the above theoretical result, we can construct the following algorithm for fuzzy clustering analysis.

**Step 1 .**

Compute the similar degree between the objects *x*_*i*_ and *x*_*j*_, *i*, *j* = 1,2,…, *n*, and then construct the fuzzy similarity matrix *R* = (*r*_*ij*_)_*n*×*n*_.

**Step 2 .**

If the fuzzy similarity matrix *R* = (*r*_*ij*_)_*n*×*n*_ is a fuzzy equivalence matrix, then we can obtain the *λ*-cutting matrix *R*_*λ*_ = (*r*_*ij*_^(*λ*)^)_*n*×*n*_ of the fuzzy equivalence matrix *R*; otherwise, we make the composition operation “∘” for the fuzzy similarity matrix *R* and derive fuzzy equivalence matrix R* by using the square method. Furthermore, we can construct the *λ*-cutting matrix *R*_λ_* = (*r*_*ij*_^∗(*λ*)^)_*n*×*n*_ of the fuzzy equivalence matrix R*.

**Step 3 .**

If all elements of the *i*th line (column) in the equivalence matrix *R*_*λ*_ (or  *R*_λ_*) are the same as the corresponding elements of the *j*th line (column) in the equivalence matrix *R*_*λ*_ (or  *R*_λ_*), then the objects *x*_*i*_ and *x*_*j*_ are of the same type, and *λ* is called as the confidence level. According to this principle, we can classify the all *n* objects *x*_*i*_ on *X* based on the different confidence level *λ*, *i* = 1,2,…, *n*; thus, we have a dynamic clustering graph.

## 3. Numerical Application

The ranking football team was the B problem of China Undergraduate Mathematical Contest in Modeling in 1993. The match points of 12 football teams in 1988-1989's Chinese National Football League are given. The match point table contains a great amount of data, and some data are missing. The details are listed in Table 1.

It is difficult to rank the 12 football teams directly according to the original data in Table 1 since the numbers of fields are in huge difference between some teams. Aimed at the amount of missing data, some researcher proposed some methods to deal with these problems. For example, Dadelo et al. [19] applied the multicriteria assessment method, TOPSIS, in ranking system of sport team. Jalao et al. [20] investigated stochastic AHP method to solve ranking and decision making problem. Keener [21] applied Perron-Frobenius theorem in ranking of football teams. In this paper, we will apply fuzzy cluster analysis to investigate such ranking problem. And firstly we approximately recognize the competency of every team through the numbers of wins, loses, and draws of fields that they attended. The data are listed in Table 2.

Then, we consider the average numbers of goals (*G*), goals against (GA), and their difference (*G* − GA) of each team in matches, which may help us to realize each team's competency. The result is listed in Table 3.

After a simple analysis of Tables 2 and 3, we can conclude that the football team *T*_7_ is the best and *T*_4_ is the worst; teams *T*_5_, *T*_6_, *T*_10_, *T*_11_, and *T*_12_ are low-ranking and *T*_1_, *T*_2_, *T*_3_, *T*_9_, and *T*_8_ are moderate in ranking. But the gap among them is not very big. Besides, considering that the data are asymmetry and incomplete, it is not very reasonable to rank the football teams just according to Tables 2 and 3.

In order to give a more reasonable and reliable ranking result, we propose a more efficient algorithm that can make full use of given data. Considering that (1) there are some teams that did not play a match with each other at all and the match points are unknown and (2) the match times between some football teams have a large difference, the number may be 0, 1, 2, and even 4. Therefore, we have to establish a rule and define a group of characteristic data for each team. Then we can compute the degrees of similarity of competencies of the 12 teams (i.e., degrees of fuzzy similarity). According to Tables 2 and 3, we propose the method of fuzzy clustering to compute the rank of the 12 teams.

### 3.1. Model Hypotheses

For conveniences, we give the following hypotheses.

1. If team *T*_*i*_ does not play with team *T*_*j*_, then we assume the only match result between *T*_*i*_ and *T*_*j*_ is *A* : *B*. Let *Q* = *A* − *B* and set *Q* = 0 in this paper.
2. Each match, every goal, and every goal against play an equally important role in ranking.
3. We only use the difference of the numbers of goals and goals against to decide the characteristic data for each team. Then the characteristic data for the *i*th team *T*_*i*_ is denoted by *r*_*i*_ = (*r*_*i*1_, *r*_*i*2_,…, *r*_*in*_) where *n* = 12,  *i* = 1,2,…, 12.

Considering that, in football fields, it is easier for team *T*_*i*_ to beat team *T*_*j*_ 2 : 1 in one match than beat *T*_*j*_ 2 : 1 in both two matches and much easier than get a 2 : 1 winning in three matches. Therefore, weighting factors shall be included when we compute characteristic data. For example, if *T*_*i*_ gets a 2 : 1 winning over *T*_*j*_, then *r*_*ij*_ = (2 − 1)*S*; if *T*_*i*_ gets 2 : 1 winning over *T*_*j*_ two times, then

(5) $$\begin{array}{c} r_{ij}=\frac{\left(2-1\right)+\left(2-1\right)}{2}V; \end{array}$$

if the winning comes to three times, then

(6) $$\begin{array}{c} r_{ij}=\frac{\left(2-1\right)+\left(2-1\right)+\left(2-1\right)}{3}U, \end{array}$$

where *U* > *V* > *S*, and set *U* = 1.4, *V* = 1.2 and *S* = 1.0 in this paper.

(4) The characteristic data for team *T*_*i*_ and itself is defined by *r*_*ii*_ = 0.
(5) The degree of fuzzy similarity between team *T*_*i*_ and *T*_*j*_ is computed by
  (7) $$\begin{array}{c} x_{ij}=1-0.038\overset{12}{\underset{k=1}{\sum }}\left|r_{ik}-r_{jk}\right|. \end{array}$$
(6) The ranking principle is that the earlier the teams cluster, the closer they are in ranking.

### 3.2. Mathematical Modeling

Suppose the universe of discourse *T* = {*T*_1_, *T*_2_, *T*_3_, *T*_4_, *T*_5_, *T*_6_, *T*_7_, *T*_8_, *T*_9_, *T*_10_, *T*_11_, *T*_12_}. According to the given data, we can obtain the following characteristic data *r*_*i*_ for the *i*th football team, *i* = 1,2,…, 12,

(8) r1=(0,0,−0.467,2.333,2,1,−1.8,−0.6,3,0,0,0),r2=(0,0,−0.467,0.933,0,1,0,0,1.2,0,0,0),r3=(0.467,0.467,0,0.933,1,3,1,−1,1,−1,0,0),r4=(−2.333,−0.933,−0.933,0,−1,   −1,−3.6,−0.6,−0.6,−0.6,0,0),r5=(−2,0,−1,1,0,−1,0,0,0,0,0,−0.6),r6=(−1,−1,−3,1,1,0,0,0,0,0,0,0),r7=(1.8,0,−1,3.6,0,0,0,1.4,2.333,2.333,2,2),r8=(0.6,0,1,0.6,0,0,−1.4,0,0,0,2,0),r9=(−3,−1.2,−1,0.6,0,0,−2.333,0,0,1.867,1,1),r10=(0,0,1,0.6,0,0,−2.333,0,−1.867,0,1,2),r11=(0,0,0,0,0,0,−2,−2,−1,−1,0,−0.467),r12=(0,0,0,0,0.6,0,−2,0,−1,−2,0.467,0).

Then we can calculate the degrees of fuzzy similarity *r*_*ij*_ between football teams *T*_*i*_ and *T*_*j*_ and obtain the fuzzy similarity matrix *R* as follows:

(9) $$\begin{array}{c} R=\left(\begin{array}{cccccccccccc} 1 & 0.666 & 0.544 & 0.351 & 0.473 & 0.496 & 0.339 & 0.514 & 0.306 & 0.346 & 0.511 & 0.526\\ & 1 & 0.641 & 0.397 & 0.65 & 0.597 & 0.389 & 0.59 & 0.351 & 0.392 & 0.587 & 0.678\\ & & 1 & 0.275 & 0.483 & 0.506 & 0.182 & 0.493 & 0.184 & 0.296 & 0.531 & 0.516\\ & & & 1 & 0.645 & 0.511 & 0.004 & 0.453 & 0.569 & 0.372 & 0.602 & 0.572\\ & & & & 1 & 0.749 & 0.313 & 0.62 & 0.574 & 0.422 & 0.577 & 0.557\\ & & & & & 1 & 0.26 & 0.567 & 0.521 & 0.369 & 0.488 & 0.549\\ & & & & & & 1 & 0.405 & 0.303 & 0.237 & 0.129 & 0.179\\ & & & & & & & 1 & 0.529 & 0.681 & 0.648 & 0.699\\ & & & & & & & & 1 & 0.478 & 0.42 & 0.471\\ & & & & & & & & & 1 & 0.572 & 0.623\\ & & & & & & & & & & 1 & 0.828\\ & & & & & & & & & & & 1 \end{array}\right). \end{array}$$

After calculating the fuzzy equivalence matrix of fuzzy similarity matrix *R* and the classification results and combining the information from Tables 2 and 3, we think that team *T*_4_ is ranked as number 12, the last place. And known by hypothesis (6), the first team clustered with *T*_4_ is *T*_5_; thus, *T*_5_ is ranked as number 11. The rest process is just similar. Team *T*_7_ is ranked as number 1 since it is the last team falling into the cluster of *T*_4_. The whole ranking result is listed in Table 4.

### 3.3. Parameter Sensitivity Analysis

There are four parameters in our model and they are predefined which seems very subjective. To make sure the ranking result is reasonable and reliable, we shall analyze whether the result will change when these parameters vary in a certain range. Some calculating results are listed in Table 5.

Known by Table 5, we can find that the ranking result does not change when *Q* ∈ [−0.3,0.3] and the absolute value of change quantity of *U*, *V*, *S* is less than 0.2, which implies our result is not sensitive to these parameters. That shows our ranking result is stable when the parameters vary slightly. Therefore, the result is reasonable and reliable.

## 4. Conclusion

In this paper, we propose the ranking algorithm based on the fuzzy clustering and obtain the ranking result as follows: *T*_7_, *T*_3_, *T*_1_, *T*_9_, *T*_10_, *T*_8_, *T*_11_, *T*_12_, *T*_2_, *T*_6_, *T*_5_, *T*_4_. Our study shows that our ranking result is reliable and stable when the parameters change in a certain range. And our algorithm can be easily generalized to the case in which the number of teams is an arbitrary positive integer *N*.

By the way, it needs to be pointed out that there are some disadvantages in our algorithm. (1) it will not rank the teams when it is difficult to figure out which teams have better or worse competition results; (2) the algorithm will fail when there are too much missing data.

## Figures and Tables

**Table 1:** Match scores of 12 football teams.

	*T*_1_	*T*_2_	*T*_3_	*T*_4_	*T*_5_	*T*_6_
*T*_1_	*X*	0 : 1, 1 : 0, 0 : 0	2 : 2, 1 : 0, 0 : 2	2 : 0, 3 : 1, 1 : 0	3 : 1	1 : 0
*T*_2_		*X*	2 : 0, 0 : 1, 1 : 3	0 : 0, 2 : 0, 0 : 0	1 : 1	2 : 1
*T*_3_			*X*	4 : 2, 1 : 1, 0 : 0	2 : 1	3 : 0
*T*_4_				*X*	2 : 3	0 : 1
*T*_5_					*X*	0 : 1
*T*_6_						*X*
*T*_7_						
*T*_8_						
*T*_9_						
*T*_10_						
*T*_11_						
*T*_12_						

	*T*_7_	*T*_8_	*T*_9_	*T*_10_	*T*_11_	*T*_12_

*T*_1_	0 : 1, 1 : 3	0 : 2, 2 : 1	1 : 0, 4 : 0	1 : 1, 1 : 1	*X*	*X*
*T*_2_	1 : 1, 1 : 1	0 : 0, 0 : 0	2 : 0, 1 : 1	0 : 2, 0 : 0	*X*	*X*
*T*_3_	1 : 0, 1 : 4	0 : 1, 3 : 1	1 : 0, 2 : 3	0 : 1, 2 : 0	*X*	*X*
*T*_4_	0 : 5, 2 : 3	2 : 1, 1 : 3	0 : 1, 0 : 0	0 : 1, 1 : 1	*X*	*X*
*T*_5_	*X*	*X*	*X*	*X*	1 : 0, 1 : 2	0 : 1, 1 : 1
*T*_6_	*X*	*X*	*X*	*X*	*X*	
*T*_7_	*X*	1 : 0, 2 : 0, 0 : 0	2 : 1, 3 : 0, 1 : 0	3 : 1, 3 : 0, 1 : 0	3 : 1	2 : 0
*T*_8_		*X*	0 : 1, 1 : 2, 2 : 0	1 : 1, 1 : 0, 0 : 1	3 : 1	0 : 0
*T*_9_			*X*	3 : 0, 1 : 0, 0 : 0	1 : 0	1 : 0
*T*_10_				*X*	1 : 0	2 : 0
*T*_11_					*X*	1 : 1, 1 : 2
*T*_12_						*X*

**Table 2:** Numbers of wins, loses, and draws.

	*T*_1_	*T*_2_	*T*_3_	*T*_4_	*T*_5_	*T*_6_	*T*_7_	*T*_8_	*T*_9_	*T*_10_	*T*_11_	*T*_12_
Wins	10	5	8	1	2	2	13	6	7	6	1	2
Loses	5	4	4	12	5	3	1	8	8	5	6	3
Draws	4	6	3	6	2	0	3	3	2	6	2	4

Total	19	15	15	19	9	5	17	17	17	17	9	9

**Table 3:** Numbers of goals, goals against, and their difference.

	*T*_1_	*T*_2_	*T*_3_	*T*_4_	*T*_5_	*T*_6_
Goals	1.412	0.8	1.333	0.632	1	0.6
Goals against	0.941	0.667	0.8	1.681	1.444	1.2
*G* − GA	0.471	0.133	0.533	−1.052	−0.444	−0.6

	*T*_7_	*T*_8_	*T*_9_	*T*_10_	*T*_11_	*T*_12_

Goals	2.059	0.911	0.647	0.882	0.778	0.667
Goals against	0.588	0.824	1	1	1.556	1
*G* − GA	1.471	0.118	−0.353	−0.118	−0.778	−0.333

**Table 4:** Ranking results of 12 football teams.

Team	*T*_1_	*T*_2_	*T*_3_	*T*_4_	*T*_5_	*T*_6_	*T*_7_	*T*_8_	*T*_9_	*T*_10_	*T*_11_	*T*_12_
Rank	3	9	2	12	11	10	1	6	4	5	7	8

**Table 5:** Some ranking results with varied parameters.

Team	*T*_1_	*T*_2_	*T*_3_	*T*_4_	*T*_5_	*T*_6_	*T*_7_	*T*_8_	*T*_9_	*T*_10_	*T*_11_	*T*_12_
*Q* = 0, *U* = 1.4, *V* = 1.2, *S* = 1	3	9	2	12	11	10	1	6	4	5	7	8
*Q* = 0.1, *U* = 1.4, *V* = 1.2, *S* = 1	3	9	2	12	11	10	1	6	4	5	7	8
*Q* = 0.2, *U* = 1.4, *V* = 1.2, *S* = 1	3	9	2	12	11	10	1	6	4	5	7	8
*Q* = 0.3, *U* = 1.4, *V* = 1.2, *S* = 1	3	9	2	12	11	10	1	6	4	5	7	8
*Q* = −0.1, *U* = 1.4, *V* = 1.2, *S* = 1	3	9	2	12	11	10	1	6	4	5	7	8
*Q* = −0.2, *U* = 1.4, *V* = 1.2, *S* = 1	3	9	2	12	11	10	1	6	4	5	7	8
*Q* = −0.3, *U* = 1.4, *V* = 1.2, *S* = 1	3	9	2	12	11	10	1	6	4	5	7	8
*Q* = 0, *U* = 1.5, *V* = 1.3, *S* = 1	3	9	2	12	11	10	1	6	4	5	7	8
*Q* = 0.1, *U* = 1.6, *V* = 1.4, *S* = 1.1	3	9	2	12	11	10	1	6	4	5	7	8
